# Gene Expression in Circulating Leukocytes in Brown Swiss and Holstein Cows During the Transition Period

**DOI:** 10.3390/ani16121858

**Published:** 2026-06-16

**Authors:** Marta Sfulcini, Vincenzo Lopreiato, Fiorenzo Piccioli-Cappelli, Luca Cattaneo, Matteo Mezzetti, Alessandro Catellani, Erminio Trevisi, Andrea Minuti

**Affiliations:** 1 Department of Animal Science, Food and Nutrition (DIANA), Faculty of Agricultural, Food and Environmental Sciences, Università Cattolica del Sacro Cuore, 29122 Piacenza, Italy; marta.sfulcini@unicatt.it (M.S.); fiorenzo.piccioli@unicatt.it (F.P.-C.); luca.cattaneo@unicatt.it (L.C.); matteo.mezzetti@unicatt.it (M.M.); alessandro.catellani@unicatt.it (A.C.); erminio.trevisi@unicatt.it (E.T.); 2 Department of Veterinary Sciences, Università di Messina, 98168 Messina, Italy; vincenzo.lopreiato@unime.it; 3 Romeo and Enrica Invernizzi Research Center for Sustainable Dairy Production of the Università Cattolica del Sacro Cuore (CREI), 29122 Piacenza, Italy

**Keywords:** dairy cow, transition period, gene expression, leukocytes, Brown Swiss, Holstein, qPCR, inflammation

## Abstract

The transition period is characterized by major metabolic and immunological changes that increase disease susceptibility in dairy cows. Genetic background may influence the regulation of immune responses during this phase. The aim of this study was to compare the expression of immune-related genes in circulating leukocytes of Brown Swiss and Holstein cows across the transition period. Twenty-two pregnant heifers (10 Brown Swiss and 12 Holstein) were managed under identical conditions and sampled at −21, +7, and +28 days relative to calving. Whole blood was collected using PAXgene tubes, and gene expression was analyzed by quantitative PCR. Target genes were associated with immune recognition, leukocyte migration and adhesion, antimicrobial activity, oxidative stress, and inflammatory response. Gene expression profiles differed between breeds over time. Holstein cows showed higher expression of genes involved in pathogen recognition before calving, including *CD14* and *TLR4*. In contrast, Brown Swiss cows showed higher expression of genes related to leukocyte migration (*ITGAL*, *CCR2*), antimicrobial activity (*MMP9*, *MPO*), inflammatory response (*TNFA*, *IL18*), and oxidative stress (*ALOX5*, *ALOX15*), particularly after calving. These results indicate breed-related differences in leukocyte gene expression and suggest that genetic background influences immune adaptation during the transition period.

## Simple Summary

The period around calving is a critical phase for dairy cows, during which animals experience major physiological changes that increase their risk of disease. Understanding how different breeds cope with this phase can help improve animal health and management. In this study, we compared two common dairy breeds, Brown Swiss and Holstein, by examining how the expression of genes related to the immune system changes before and after calving. Blood samples were collected at different time points, and differences between breeds were evaluated. The results showed that the two breeds display different patterns of gene activity. Holstein cows showed higher activity of genes involved in early detection of potential threats before calving, whereas Brown Swiss cows showed higher activity of genes associated with immune cell movement and defense mechanisms after calving. These findings indicate that genetic differences between breeds influence how cows adapt to this critical phase and may help develop strategies to improve animal health and resilience.

## 1. Introduction

The transition period is the most critical phase of the lactation cycle and is characterized by major metabolic, endocrine, and immunological changes. During this phase, dairy cows experience negative energy balance, increased lipid mobilization, oxidative stress, and systemic inflammatory responses, all of which contribute to a temporary impairment of immune function [1]. The sequence and interaction of metabolic, oxidative, and inflammatory events leading to immune dysfunction during the transition period are still not fully understood, and it remains unclear which mechanisms primarily drive periparturient immune dysregulation. Polymorphonuclear neutrophils (PMN) and lymphocyte functions progressively decline starting approximately two weeks before calving, reaching their lowest efficiency around parturition and in the early postpartum period [2,3,4]. This reduced immune competence is associated with increased susceptibility to infectious and metabolic diseases, ultimately affecting milk production, reproductive performance, longevity, and animal welfare.

Genetic background may play a key role in modulating the immune and inflammatory responses during the transition period. Breeds selected for different production traits have been shown to exhibit distinct physiological and molecular responses to peripartum challenges, including differences in immune-related gene expression and the regulation of innate and adaptive immunity between Simmental and Holstein cows [5].

Over the last few decades, dairy cattle breeding has strongly focused on improving milk yield and composition. Consequently, breeds characterized by different breeding goals and production aptitudes, such as Holstein and Brown Swiss, may also differ in physiological adaptation mechanisms during the transition period [6]. Holstein cows have been primarily selected for high milk yield, whereas Brown Swiss cows have shown improvements in milk quality traits, particularly those related to cheese production [7]. Despite these known differences, limited information is available on how these two breeds differ in terms of immune-related gene expression during the transition period. Understanding these differences could provide new insights into breed-specific strategies for coping with metabolic and immunological challenges.

Given the physiological and productive differences between Holstein and Brown Swiss cows, we hypothesized that the two breeds would exhibit distinct patterns of immune-related gene expression during the transition period, reflecting differences in adaptation to periparturient challenges. Therefore, the aim of this study was to compare the expression of immune-related genes in circulating leukocytes of Brown Swiss and Holstein cows across the transition period, in order to evaluate potential differences in immune adaptation between breeds.

## 2. Materials and Methods

### 2.1. Animals, Treatments, and PAXgene Blood Sampling

The trial was conducted in accordance with Italian legislation on animal experimentation (DL n. 26, 4 March 2014) and ethical standards (Authorization of Italian Health Ministry N 403/2017-PR). The experimental design included 10 pregnant Brown Swiss heifers (BS; *n* = 10) and 12 pregnant Holstein heifers (HS; *n* = 12), homogeneous in terms of age and calving period. Following calving, the same animals were evaluated during the postpartum period. These animals calved between October 2019 and May 2020. Management procedures for both breeds were kept identical to minimize any external factors unrelated to genotype. All animals included in the study underwent normal calving without dystocia or major postpartum disorders. Body condition score (BCS) was monitored throughout the experimental period. Before calving, BCS was 3.58 ± 0.08 and 3.52 ± 0.10 for Brown Swiss and Holstein cows, respectively, whereas postpartum BCS was 3.19 ± 0.12 and 2.91 ± 0.23, respectively. All animals were managed according to the farm vaccination protocol and were vaccinated with Bovigen^®^ (Virbac, Carros, France) 30 days before calving.

The cows were housed together during the late pregnancy period (from the seventh month of gestation until calving) and throughout lactation and were housed in individual tied stalls with controlled environmental conditions. They were fed a total mixed ration (Table 1).

Blood samples were collected 21 days before calving, and 7 and 28 days after calving, from the jugular vein using PAXgene Blood System tubes (Preanalytix, Hombrechtikon, Switzerland) for RNA extraction. The use of PAXgene tubes in cattle has been previously reported [5,9,10]. Simultaneously, blood samples were collected in K-EDTA tubes for hematological profile analysis (Cell-DYN 3700, Abbott Diagnostic Division, Santa Clara, CA, USA). K-EDTA tubes were immediately sent to Istituto Zooprofilattico Sperimentale della Lombardia e dell’Emilia-Romagna “Bruno Umbertini” (Strada della Faggiola, 1, 29027, Gariga, PC, Italy) and analyzed within 2 h of collection for assessing complete blood count (automate haematology analyser for veterinary use XN-V-1000, Sysmex, Kobe, Japan). Parameters considered in the current study were total white blood cells (WBC) count and the WBC differential counts for neutrophils, eosinophils, basophils, lymphocytes, and monocytes.

### 2.2. RNA Extraction, cDNA Synthesis, Target Genes, Quantitative PCR

Total RNA was extracted from whole blood samples collected in PAXgene Blood RNA tubes (PreAnalytix GmbH, Qiagen, Hilden, Germany) according to the manufacturer’s instructions and as previously described [11]. RNA concentration was determined using the Qubit RNA BR Assay Kit (Invitrogen, Thermo Fisher Scientific, Waltham, MA, USA), and RNA integrity was assessed using the Experion Automated Electrophoresis System (Bio-Rad, Hercules, CA, USA). Each RNA sample was diluted to a final concentration of 100 ng/μL using nuclease-free water prior to reverse transcription. Synthesis of cDNA was performed using a reverse transcription kit (RevertAid RT Reverse Transcription Kit; Thermo Fisher Scientific, Waltham, MA, USA). The resulting cDNA was diluted 1:4 (vol/vol) in DNase/RNase-free water and stored at −80 °C until analysis. Quantitative PCR (qPCR) was performed as previously described by Florida et al. [12] using a CFX384 Touch Real-Time PCR Detection System (Bio-Rad, Hercules, CA, USA). Each reaction (10 μL) consisted of 4 μL of diluted cDNA and 6 μL of reaction mixture containing 1 × SYBR Green Master Mix (Applied Biosystems, Warrington, UK), 0.4 μL of each forward and reverse primer (10 μM), and nuclease-free water. All samples were analyzed in triplicate. The target genes selected for transcript analysis were related to immune recognition and media-tion (*CD14*, *CD16*, *MYD88*, *TLR4*, *TLR2*), cell migration and adhesion (*CCR2*, *CX3CR1*, *ITGB2*, *ITGAL*, *SELL*, *SELPLG*, *CD44*, *VCAM1*, *IL8*), antimicrobial functions (*MMP9*, *MPO*, *LCN2*, *LYZ*, *PRTN3*, *IDO1*), oxidative stress and leukotriene function (*ALOX5*, *ALOX15*, *SOD1*, *SOD2*, *NOS2*), and inflammatory response (*CASP1*, *TNFRSF1A*, *IL6R*, *IL10*, *TNFA*, *IL6*, *IL1B*, *IL1R*, *IRAK1*, *IL18*, *NLRP3*, *S100A8*). Primer sequences and amplicon size are reported in Supplementary Table S1. Primer specificity was confirmed by melting curve analysis, and only reactions showing a single peak were considered for analysis. Amplicon size was verified by agarose gel electrophoresis. Quantification cycle (Cq) values and amplification efficiencies were calculated using LinRegPCR software (version 2017.1). PCR amplification efficiencies ranged from 90.7% to 101.1% (Supplementary Table S1). Gene expression data were normalized using the geometric mean of three internal control genes (*ACTB*, *SDHA*, and *YWHAZ*). The stability of the normalization factor was assessed using GeNorm, yielding a pairwise variation of 0.11. No further improvement in stability was observed by including an additional reference gene.

### 2.3. Statistical Analysis

Statistical analysis was performed using ANOVA in the MIXED procedure of SAS Version 9.4 (SAS Institute Inc., Cary, NC, USA) according to the following model:

Yijw = μ + Ti + Dj + TDij + Sw + εijw

where Yijw = dependent variable; μ = total mean; Ti = fixed effect of breed (i = BS vs. HS); Dj = fixed effect of day from calving (j = −21, +7, and +28 day from calving); TDij = interaction between breed and day of lactation; Sw = random effect of the animal (cow) nested within breed × days relative to calving; and εijkl = residual error.

The Kenward–Roger statement was used for computing the denominator degrees of freedom. The normality of the data was checked with the Univariate procedure of SAS (ver. 9.4). Variables not normally distributed were log10 transformed. Asterisks in tables with results indicate variables that were transformed. Data are reported as least squares means (LSM) ± mean standard error (SEM). Comparisons between groups with *p* values less than 0.05 were reported as statistically significant. Those with values between 0.1 and 0.05 were discussed in terms of trend.

## 3. Results

**Figure 1:**
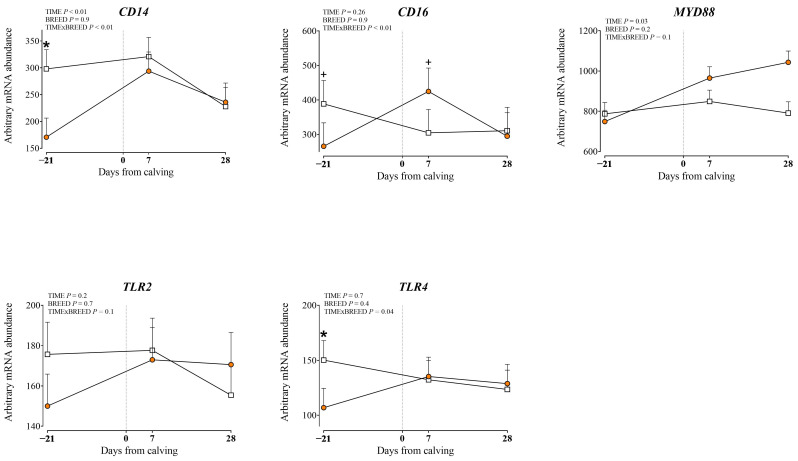
Arbitrary mRNA abundance for genes related to recognition and immune mediation functions in whole blood leukocytes of Brown Swiss (BS, *n* = 10; orange circles) and Holstein (HS, *n* = 12; white squares) cows at −21, +7, and +28 days relative to calving. Data are presented as least squares means (LSM) and SEM. Significance levels of the main effects of the model are reported. * Indicates a significant difference (*p* ≤ 0.05), + indicates a trend (0.05 < *p* ≤ 0.10).

**Figure 2:**
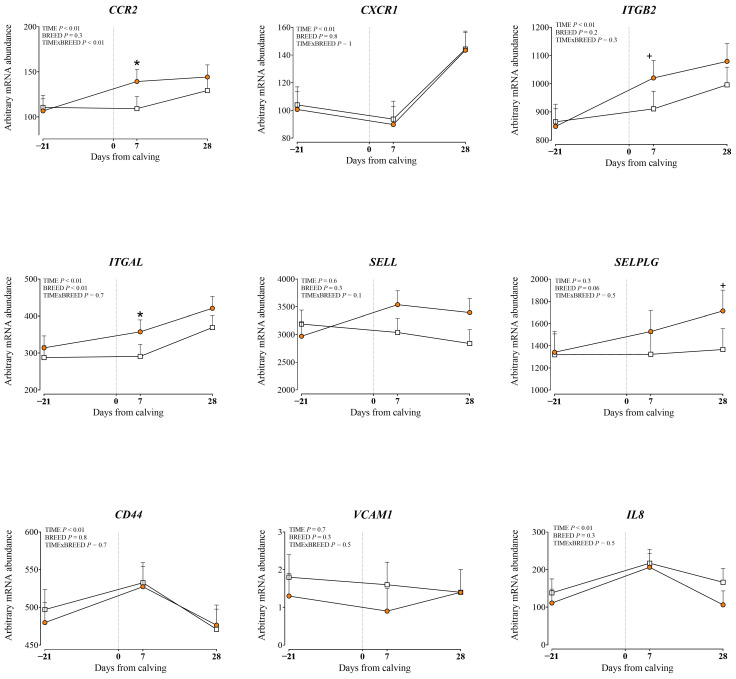
Arbitrary mRNA abundance for genes related to migration and cell adhesion functions in whole blood leukocytes of Brown Swiss (BS, *n* = 10; orange circles) and Holstein (HS, *n* = 12; white squares) cows at −21, +7, and +28 days relative to calving. Data are presented as least squares means (LSM) and SEM. Significance levels of the main effects of the model are reported. * Indicates a significant difference (*p* ≤ 0.05), + indicates a trend (0.05 < *p* ≤ 0.10).

**Figure 3:**
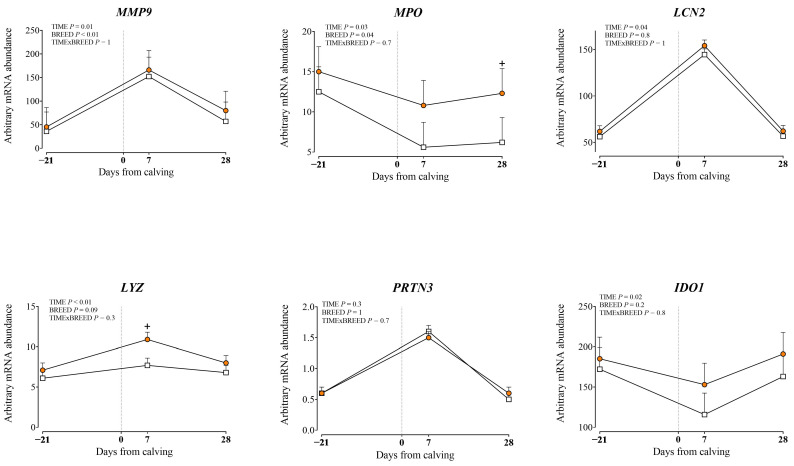
Arbitrary mRNA abundance for genes related to antimicrobial strategies in whole blood leukocytes evaluated in Brown Swiss (BS, *n* = 10; orange circles) and Holstein (HS, *n* = 12; white squares) cows at −21, +7, and +28 days relative to calving. Data are presented as least squares means (LSM) and SEM. Significance levels of the main effects of the model are reported. + indicates a trend (0.05 < *p* ≤ 0.10).

**Figure 4:**
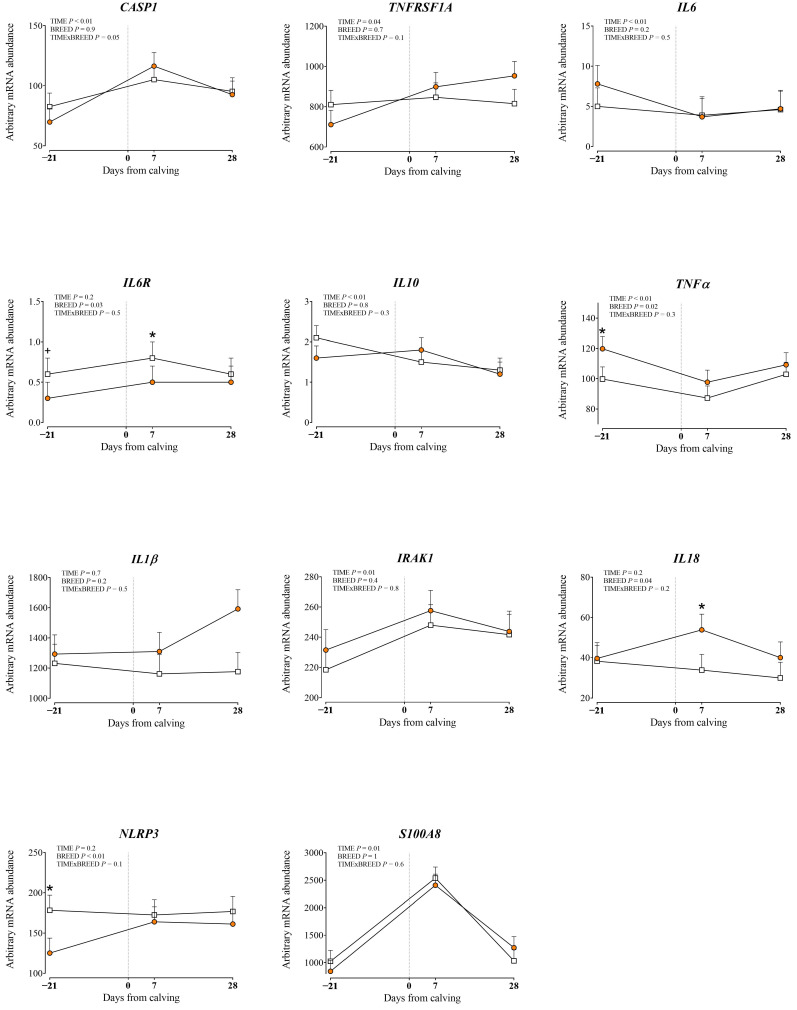
Arbitrary mRNA abundance for genes related to the inflammatory cascade in whole blood leukocytes evaluated in Brown Swiss (BS, *n* = 10; orange circles) and Holstein (HS, *n* = 12; white squares) cows at −21, +7, and +28 days relative to calving. Data are presented as least squares means (LSM) and SEM. Significance levels of the main effects of the model are reported. * Indicates a significant difference (*p* ≤ 0.05), + indicates a trend (0.05 < *p* ≤ 0.10).

**Figure 5:**
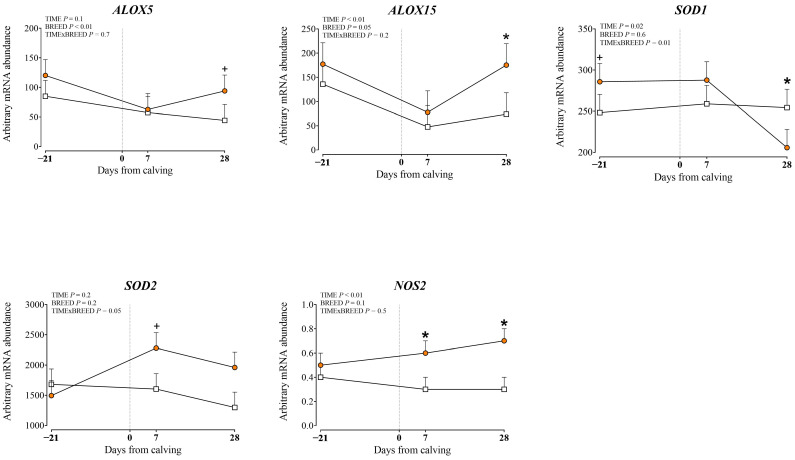
Arbitrary mRNA abundance for genes related to oxidative stress in whole blood leukocytes evaluated in Brown Swiss (BS, *n* = 10; orange circles) and Holstein (HS, *n* = 12; white squares) cows at −21, +7, and +28 days relative to calving. Data are presented as least squares means (LSM) and SEM. Significance levels of the main effects of the model are reported. * Indicates a significant difference (*p* ≤ 0.05), + indicates a trend (0.05 < *p* ≤ 0.10).

Genes were grouped into functional pathways (Figure 1, Figure 2, Figure 3, Figure 4 and Figure 5) and reported as least-squares means (LSM) of arbitrary mRNA abundance with the standard error of the mean (SEM). The effects of breed (BS; HS), days from calving (time), and their interaction are reported.

### 3.1. Expression of Genes Involved in the TLR Pathway and Immune Mediation

The mRNA abundance of genes involved in the TLR pathway and immune mediation is shown in Figure 1.

*CD14*, *TLR4*, and *CD16* expression were affected by the interaction between breed and days from calving (*p* < 0.05). HS cows showed greater expression than BS cows at −21 days from calving, whereas for *CD16*, an opposite tendency was observed at +7 days. *MYD88* and *TLR2* were influenced by days from calving (*p* < 0.03), showing increased expression after calving, with no differences between breeds.

### 3.2. Expression of Genes Involved in Cell Migration and Adhesion

The mRNA abundance of genes involved in cell migration and adhesion is shown in Figure 2.

*CCR2* expression was affected by the interaction between breed and days from calving (*p* < 0.01), with greater expression in BS cows at +7 days from calving. *ITGAL* expression differed between breeds (*p* < 0.01) and was also influenced by days from calving (*p* < 0.01), with greater expression in BS cows and increased values after calving. *ITGB2* and *SELPLG* showed a similar pattern, with higher values in BS cows, particularly around +7 and +28 days (*p* < 0.10). *CD44* and *IL8* were affected by days from calving (*p* < 0.01), showing a common temporal pattern characterized by higher values around +7 days, with no differences between breeds.

### 3.3. Expression of Genes Involved in Antimicrobial Functions

The mRNA abundance of genes involved in antimicrobial functions is shown in Figure 3.

*MMP9* and *MPO* showed greater expression in BS cows (*p* < 0.05), with *MPO* differences more evident at +28 days. *LCN2* expression was affected by days from calving (*p* < 0.01), showing higher values around +7 days. In contrast, *IDO1* showed lower overall expression (*p* < 0.05) and an opposite temporal pattern, decreasing at the same time point. *LYZ* showed a tendency for greater expression in BS cows, particularly around +7 days (*p* < 0.10).

### 3.4. Expression of Genes Involved in the Inflammatory Response

The mRNA abundance of genes involved in the inflammatory response is shown in Figure 4.

*NLRP3* and *IL6R* expression differed between breeds *(p* < 0.05), with greater expression in HS cows, particularly before calving and in early postpartum. *IL18* and *TNFA* showed greater expression in BS cows (*p* < 0.05), with *TNFA* differences more evident at −21 days from calving. *CASP1*, *IL6*, *IL10*, and *IL1B* were affected by days from calving (*p* < 0.01), showing increased expression after calving, with peaks around +7 days. *IL1R* showed a tendency for greater expression in BS cows at +28 days (*p* < 0.10).

### 3.5. Expression of Genes Involved in Oxidative Stress and Leukotriene Pathways

The mRNA abundance of genes involved in oxidative stress and leukotriene pathways is shown in Figure 5.

*ALOX5* and *ALOX15* showed greater expression in BS cows (*p* ≤ 0.05), with *ALOX15* differences more evident at +28 days. *SOD2* showed greater expression in BS cows (*p* < 0.05) and was also influenced by days from calving, with increased values after calving. *SOD1* expression was affected by the interaction between breed and days from calving (*p* = 0.01), with BS cows showing higher values before calving and HS cows at +28 days. *NOS2* was affected by days from calving (*p* < 0.01), showing increased expression after calving, with no differences between breeds.

### 3.6. Hematological Profile

The results of the hematological profile are shown in Table 2.

A significant difference between breeds for total white blood cell count (*p* = 0.05): the Holstein cows have higher concentrations. At day −21, the percentage of neutrophils (*p* = 0.03), monocytes (*p* < 0.01), basophils (*p* = 0.02), and the total count (*p* < 0.01) are higher in the Holstein; for the basophils during the whole sampling period, the Holstein resulted in significantly higher values. The only major parameter in the brown breed was the lymphocytes at −21 days (*p* = 0.04).

## 4. Discussion

The transition period represents a critical phase for dairy cows, characterized by major physiological, metabolic, and immunological adaptations required to support the onset of lactation. During this phase, cows undergo profound changes in nutrient partitioning and endocrine regulation [13], while a mismatch between nutrient intake and increasing energy demands often leads to the development of negative energy balance and mobilization of body reserves [14].

In parallel, several studies have documented a reduction in immune competence, with decreased functionality of polymorphonuclear cells and lymphocytes from approximately two weeks before calving through early postpartum [15]. This condition is associated with impaired phagocytic activity, reduced leukocyte migration and adhesion, and decreased antimicrobial capacity. In addition, the transition period is characterized by a complex interaction between metabolic and immune pathways, often accompanied by a transient inflammatory state [16], which may contribute to increased susceptibility to disorders during early lactation.

Calving is also associated with increased exposure to pathogens, particularly at the uterine level, requiring a rapid activation of the innate immune response. In the present study, *TLR4*, *CD14*, and *CD16* expression showed a significant interaction between breed and days from calving, with higher expression observed in HS cows at −21 days from calving. This pattern may suggest a greater basal activation of innate immune recognition and pro-inflammatory pathways before calving in this breed [17,18]. However, after calving, the expression of these genes tended to decrease in HS cows, whereas BS cows showed a different temporal pattern, characterized by increased postpartum *TLR4* expression. A similar reduction in *TLR4* expression after calving has been previously described by Crookenden et al. [19]. Toll-like receptors (TLRs), and especially *TLR4*, play a key role in recognizing pathogen-associated molecular patterns and activating downstream signaling pathways such as NF-κB, leading to the transcription of pro-inflammatory cytokines [20]. Therefore, the different temporal modulation of these genes between breeds may reflect distinct regulation of innate immune activation and inflammatory adaptation across the transition period. These transcriptional differences were also partially supported by the hematological profile, which showed breed-related differences in leukocyte populations, particularly for neutrophils, monocytes, and basophils. In general, HS cows exhibited higher circulating leukocyte counts, suggesting that transcriptional and cellular responses may follow different regulatory patterns during the transition period.

Leukocyte migration from the bloodstream to peripheral tissues represents a key component of the innate immune response, and this process is known to be impaired around calving, with reduced efficiency of leukocyte trafficking and diapedesis during early postpartum [19]. In the present study, several genes involved in leukocyte migration and adhesion showed differences between breeds and across days from calving. In particular, *ITGAL*, *ITGB2*, and *SELPLG* showed greater expression in BS cows during the transition period, supporting the involvement of leukocyte adhesion and migration pathways in breed-related immune adaptation. Since these genes are involved in leukocyte adhesion to the endothelium and subsequent transmigration into tissues, their coordinated modulation may reflect differences in the regulation of immune cell trafficking between breeds [21]. Efficient recruitment of immune cells to target tissues is crucial for the resolution of infections, and alterations in adhesion and migration pathways have been associated with increased susceptibility to disease [22,23]. During the transition period, tissues such as the mammary gland and uterus are likely to require a high recruitment of immune cells from the bloodstream. Therefore, the greater expression of these genes in BS cows, particularly during postpartum, may suggest differences in the regulation of leukocyte recruitment and activation processes between breeds.

Consistent with the hypothesis of enhanced leukocyte recruitment mechanisms in BS cows during postpartum, *CCR2* expression showed a significant interaction between breed and days from calving, with greater expression in BS cows at +7 days. Given the role of this chemokine receptor in monocyte recruitment, this result may indicate a temporally specific modulation of immune cell migration in the early postpartum phase. In contrast, *CD44* and *IL8* expression were mainly influenced by days from calving, showing a similar temporal pattern characterized by higher values around +7 days, with no differences between breeds. This common pattern may reflect a generalized activation of inflammatory and chemotactic pathways during early postpartum, independent of breed.

Genes involved in antimicrobial activity play a central role in the innate immune response, and alterations in immune function during the transition period may impair the ability of leukocytes to effectively eliminate pathogens. In the present study, *MMP9* expression was greater in BS cows, which is consistent with the expression patterns previously observed for genes involved in leukocyte adhesion and migration. Since *MMP9* plays an important role in facilitating neutrophil migration and tissue infiltration [24], its greater expression in BS cows may support the hypothesis of a more pronounced activation of antimicrobial and leukocyte recruitment mechanisms in this breed during postpartum. Similarly, *MPO*, a key enzyme involved in the intracellular killing of pathogens, showed greater expression in BS cows, particularly during the postpartum period [25]. The coordinated increase of *MMP9* and *MPO* expression in BS cows during early lactation may therefore suggest breed-related differences in the regulation of neutrophil-mediated antimicrobial responses. However, these results should be interpreted cautiously, as gene expression does not necessarily reflect enzymatic activity.

The regulation of inflammatory pathways represents a key component of the immune response during the transition period, when a balance between activation and control of inflammation is essential to maintain homeostasis [15]. In the present study, *CASP1* and *IL18* showed a similar temporal pattern, characterized by increased expression around +7 days from calving, with higher values observed in BS cows. Similarly, *NLRP3*, a key component of the inflammasome complex, also showed increased expression during early postpartum, particularly in BS cows. Since these genes are directly involved in inflammasome activation and in the maturation of pro-inflammatory cytokines, their coordinated modulation may suggest a greater activation of inflammasome-related pathways in BS cows during early lactation. This interpretation is consistent with the enhanced inflammatory responses typically observed during the postpartum period and with the temporal dynamics observed for genes involved in pathogen recognition and innate immune activation. The analysis of cytokine-related genes further supports the presence of a regulated inflammatory response. In particular, *TNFA* and its receptor *TNFRSF1A* showed opposite expression trends, which may reflect mechanisms aimed at preventing an excessive pro-inflammatory response. A similar pattern was observed for *IL6* and *IL6R*, suggesting a coordinated modulation between cytokines and their receptors. Although *IL1R* did not show significant changes, its expression pattern appeared to be consistent with that of related signaling components, including *MYD88*, suggesting a coordinated regulation within the inflammatory signaling cascade.

Genes related to lipid mediators contribute to the coordination of immune cell recruitment and activation. In the present study, *ALOX5* and *ALOX15* showed increased expression after calving, with higher values observed in BS cows. Given their role in leukotriene production and in the regulation of inflammatory processes [26,27,28], their coordinated upregulation during postpartum may suggest differences in the modulation of lipid-mediated inflammatory pathways between breeds. The temporal pattern observed for both genes, characterized by increased expression around +7 days from calving, is also consistent with the activation of inflammatory responses typically occurring during early lactation. Similarly, genes involved in oxidative stress responses showed modulation across the transition period. In particular, *SOD2* expression was greater in BS cows and increased after calving, suggesting a potential involvement in the regulation of oxidative balance during this phase. Although *SOD1* and *NOS2* were mainly influenced by days from calving, their temporal patterns support the existence of a generalized activation of oxidative stress-related pathways during early lactation.

Although gene expression analysis provides useful information regarding transcriptional adaptation during the transition period, transcript abundance does not necessarily reflect protein expression or functional immune activity. Therefore, further studies integrating protein-level measurements and functional immune assays are needed to better characterize breed-specific immune adaptation mechanisms during the transition period.

## 5. Conclusions

The transition period is characterized by profound metabolic and immunological changes that influence the regulation of immune function in dairy cows. The present study showed that Brown Swiss and Holstein cows exhibit different patterns of immune-related gene expression during the transition period, particularly during early postpartum. In general, Brown Swiss cows showed greater expression of several genes involved in leukocyte migration, antimicrobial activity, inflammasome activation, and inflammatory responses after calving. These transcriptional differences were accompanied by variations in hematological parameters, suggesting breed-related differences in immune adaptation during the transition period. Further studies integrating functional, metabolic, and productive data are needed to better clarify the biological significance of these differences.

## Figures and Tables

**Table 1:** Nutrient composition (% of DM unless otherwise noted) of TMR fed during the study period.

Item	Lactation	Dry Period
Ingredients (% of DM)		
Corn silage	32.6	11.6
Alfalfa hay	24.9	—
Corn ground	13.8	—
Soybean meal	11.4	4.8
Barley ground	9.2	—
Wheat silage	3.3	47.5
Sunflower meal	2.0	5.1
Mineral and vitamin premix	1.9	0.9
Hydrogenated fat	0.9	—
Straw	—	17.7
Ryegrass hay	—	12.4
Chemical composition		
NEL, Mcal/kg	1.65	1.28
CP, % of DM	16.6	12.7
NSC, % of DM	30.0	9.7
NDF, % of DM	33.4	57.0
Ca, % of DM	0.79	0.45
P, % of DM	0.42	0.34
DCAD, mEq/100 g of DM ^1^		+20

^1^ DCAD = (0.15 Ca + 0.15 Mg + Na + K) − (Cl^−^ + 0.6 S + 0.5 P); ([8]).

**Table 2:** Hematological profile of Brown Swiss (BS) and Holstein (HS) at 21 days before calving, 7 days after, and 28 days after calving.

		DFC				*p*-Value		
Variable	*BREED*	−21	7	28	*SEM*	*DFC*	*BREED*	*DFC* * *BREED*
NEUTROPHILS	BS	2.86 *	2.69	4.04	0.69	0.14	0.14	0.42
	HS	4.25	3.47	4.24				
pNEUTROPHILS	BS	37.93 *	37.23	45.84	4.42	0.28	0.32	0.33
	HS	44.89	41.54	43.61				
LYMPHOCYTES	BS	3.76	2.86 ^+^	3.23	0.30	<0.01 *	0.20	0.04 *
	HS	3.80	3.47	3.65				
pLYMPHOCYTES	BS	49.74 *	47.15	40.65	3.48	0.21	0.10	0.07 ^+^
	HS	40.21	43.42	41.79				
MONOCYTES	BS	0.62 *	0.81 *	0.79 *	0.07	0.12	<0.01 *	0.19
	HS	1.00	1.02	0.99				
pMONOCYTES	BS	8.21 *	13.54	10.53	1.25	<0.01 *	0.27	0.26
	HS	10.56	13.21	11.35				
EOSINOPHILS	BS	0.28	0.12	0.21	0.08	<0.01 *	0.68	0.53
	HS	0.35	0.09	0.23				
pEOSINOPHILS	BS	3.72	1.60	2.43	0.83	<0.01 *	0.75	0.76
	HS	3.71	1.07	2.41				
BASOPHILS	BS	0.03 *	0.03 *	0.04 *	0.01	<0.01 *	<0.01 *	0.98
	HS	0.06	0.06	0.08				
pBASOPHILS	BS	0.41 *	0.48 *	0.55 *	0.09	0.02 *	<0.01 *	0.77
	HS	0.63	0.77	0.84				
WBC	BS	7.55 *	6.50	8.31	0.86	0.01 *	0.05 *	0.62
	HS	9.45	8.11	9.19				

* Significant differences (*p*-value ≤ 0.05); ^+^ Differences tend to be significant (*p*-value from 0.05 to 0.1).

## Data Availability

The data presented in this study are not publicly available due to privacy and confidentiality restrictions.

## Supplementary Materials

The following supporting information can be downloaded at https://www.mdpi.com/article/10.3390/ani16121858/s1: Table S1: GenBank accession number, primer hybridization position, primer sequence, amplicon size, and amplification efficiency of primer pairs used for qPCR analysis of gene expression in Bos taurus.

## Abbreviations

The following abbreviations are used in this manuscript:

BS	Brown Swiss
HS	Holstein
DFC	days from calving
DIM	days in milk
WBC	white blood cells
pWBC	percentage of white blood cells
NEFA	nonesterified fatty acids
BHB	beta-hydroxybutyrate
qPCR	quantitative polymerase chain reaction
Cq	quantification cycle
SEM	standard error of the mean
LSM	least squares means
TMR	total mixed ration
EDTA	ethylenediaminetetraacetic acid

## References

[1] Trevisi E., Minuti A. (2018). Assessment of the Innate Immune Response in the Periparturient Cow. Res. Vet. Sci..

[2] Batistel F., Arroyo J.M., Garces C.I.M., Trevisi E., Parys C., Ballou M.A., Cardoso F.C., Loor J.J. (2018). Ethyl-Cellulose Rumen-Protected Methionine Alleviates Inflammation and Oxidative Stress and Improves Neutrophil Function during the Periparturient Period and Early Lactation in Holstein Dairy Cows. J. Dairy Sci..

[3] Kehrli M.E., Goff J.P. (1989). Periparturient Hypocalcemia in Cows: Effects on Peripheral Blood Neutrophil and Lymphocyte Function. J. Dairy Sci..

[4] Minuti A., Jahan N., Lopreiato V., Piccioli-Cappelli F., Bomba L., Capomaccio S., Loor J.J., Ajmone-Marsan P., Trevisi E. (2020). Evaluation of Circulating Leukocyte Transcriptome and Its Relationship with Immune Function and Blood Markers in Dairy Cows during the Transition Period. Funct. Integr. Genom..

[5] Lopreiato V., Minuti A., Morittu V.M., Britti D., Piccioli-Cappelli F., Loor J.J., Trevisi E. (2020). Short Communication: Inflammation, Migration, and Cell-Cell Interaction-Related Gene Network Expression in Leukocytes Is Enhanced in Simmental Compared with Holstein Dairy Cows after Calving. J. Dairy Sci..

[6] Brito L.F., Bedere N., Douhard F., Oliveira H.R., Arnal M., Peñagaricano F., Schinckel A.P., Baes C.F., Miglior F. (2021). Review: Genetic Selection of High-Yielding Dairy Cattle toward Sustainable Farming Systems in a Rapidly Changing World. Animal.

[7] De Marchi M., Bittante G., Dal Zotto R., Dalvit C., Cassandro M. (2008). Effect of Holstein Friesian and Brown Swiss Breeds on Quality of Milk and Cheese. J. Dairy Sci..

[8] Goff J.P., Horst R.L. (2003). Role of Acid-Base Physiology on the Pathogenesis of Parturient Hypocalcaemia (Milk Fever)—The DCAD Theory in Principal and Practice. Acta Vet. Scand. Suppl..

[9] Sandri M., Stefanon B., Loor J.J. (2015). Transcriptome Profiles of Whole Blood in Italian Holstein and Italian Simmental Lactating Cows Diverging for Genetic Merit for Milk Protein. J. Dairy Sci..

[10] Pošćić N., Montanari T., D’Andrea M., Licastro D., Pilla F., Ajmone-Marsan P., Minuti A., Sgorlon S. (2017). Breed and Adaptive Response Modulate Bovine Peripheral Blood Cells’ Transcriptome. J. Anim. Sci. Biotechnol..

[11] Cattaneo L., Mezzetti M., Lopreiato V., Piccioli-Cappelli F., Trevisi E., Minuti A. (2021). Gene Network Expression of Whole Blood Leukocytes in Dairy Cows with Different Milk Yield at Dry-Off. PLoS ONE.

[12] Floridia V., Sfulcini M., D’Alessandro E., Cattaneo L., Mezzetti M., Liotta L., Trevisi E., Lopreiato V., Minuti A. (2023). Effect of Different Anticoagulant Agents on Immune-Related Genes in Leukocytes Isolated from the Whole-Blood of Holstein Cows. Genes.

[13] Bauman D.E., Bruce Currie W. (1980). Partitioning of Nutrients During Pregnancy and Lactation: A Review of Mechanisms Involving Homeostasis and Homeorhesis. J. Dairy Sci..

[14] Drackley J.K. (1999). Biology of Dairy Cows During the Transition Period: The Final Frontier?. J. Dairy Sci..

[15] Trevisi E., Cattaneo L., Piccioli-Cappelli F., Mezzetti M., Minuti A. (2025). International Symposium on Ruminant Physiology: The Immunometabolism of Transition Dairy Cows from Dry-off to Early Lactation—Lights and Shadows. J. Dairy Sci..

[16] Bradford B.J., Yuan K., Farney J.K., Mamedova L.K., Carpenter A.J. (2015). Invited Review: Inflammation during the Transition to Lactation: New Adventures with an Old Flame. J. Dairy Sci..

[17] Heiser A., LeBlanc S.J., McDougall S. (2018). Pegbovigrastim Treatment Affects Gene Expression in Neutrophils of Pasture-Fed, Periparturient Cows. J. Dairy Sci..

[18] Kumar H., Kawai T., Akira S. (2011). Pathogen Recognition by the Innate Immune System. Int. Rev. Immunol..

[19] Crookenden M.A., Heiser A., Murray A., Dukkipati V.S.R., Kay J.K., Loor J.J., Meier S., Mitchell M.D., Moyes K.M., Walker C.G. (2016). Parturition in Dairy Cows Temporarily Alters the Expression of Genes in Circulating Neutrophils. J. Dairy Sci..

[20] Taraktsoglou M., Szalabska U., Magee D.A., Browne J.A., Sweeney T., Gormley E., MacHugh D.E. (2011). Transcriptional Profiling of Immune Genes in Bovine Monocyte-Derived Macrophages Exposed to Bacterial Antigens. Vet. Immunol. Immunopathol..

[21] Springer T.A. (1994). Traffic Signals for Lymphocyte Recirculation and Leukocyte Emigration: The Multistep Paradigm. Cell.

[22] Burvenich C., Paape M.J., Hill A.W., Guidry A.J., Miller R.H., Heyneman R., Kremer W.D.J., Brand A. (1994). Role of the Neutrophil Leucocyte in the Local and Systemic Reactions during Experimentally Induced *E. coli* mastitis in Cows Immediately after Calving. Vet. Q..

[23] Schmidt S., Moser M., Sperandio M. (2013). The Molecular Basis of Leukocyte Recruitment and Its Deficiencies. Mol. Immunol..

[24] Faurschou M., Borregaard N. (2003). Neutrophil Granules and Secretory Vesicles in Inflammation. Microbes Infect..

[25] Lopreiato V., Minuti A., Britti D., Perri C., Loor J.J., Trevisi E. (2019). Post-Calving Leukocyte Immune-Related Genes Are Enhanced in Simmental Compared with Holstein Cows. J. Dairy Sci..

[26] Bottons G.D., Adams H.R. (1992). Involvement of Prostaglandins and Leukotrienes in the Pathogenesis of Endotoxemia and Sepsis. J. Am. Vet. Med. Assoc..

[27] Williams K.I., Higgs G.A. (1988). Eicosanoids and Inflammation. J. Pathol..

[28] Zipser R.D., Laffi G. (1985). Prostaglandins, Thromboxanes and Leukotrienes in Clinical Medicine. West. J. Med..

