# Supplementary material for: Gene Expression in Circulating Leukocytes in Brown Swiss and Holstein Cows During the Transition Period
Source: Animals (Basel). 2026 Jun 16;16(12):1858. doi: 10.3390/ani16121858 (PMC13295243; doi:10.3390/ani16121858)
Supplement: Supplementary file 1 [file animals-16-01858-s001.zip › animals-4317637-supplementary.pdf]

Supplemental Table S1. GenBank accession number, primer hybridization position, primer sequence, amplicon size, and amplification efficiency of primer pairs used for qPCR analysis of gene expression in Bos taurus.

Accession no.	Gene	Primers[1]	Primers (5'-3')	(bp)[2]	Efficiency (%)
NM_198221.2	*ITGAL*	F.1582	ATCAACGGGGATGAGCTGAC	126	91.2
		R.1707	GGTCCCTTCTATCCGCTGAC		
XM_015459671.1	*CCR2*	F.138	CGTGGGACAAATCGAAGCAC	199	92.3
		R.336	AGCATAGTGAGCCCAGAACG		
NM_001076799.1	*NOS2*	F.1690	GCCTTCAACCCCAAGGTTCT	102	90.7
		R.1791	GTCTCCGTTGCCAAAAGTGC		
NM_001206735.1	*IL1R*	F.897	CCCGGGCGATAAAGCTGATT	104	96.4
		R.1000	AAGCCAGGATCCCAAGACCA		
NM_175781.1	*ITGB2*	F.1231	GACACCCTGAAAGTCACCTACGA	108	92.1
		R.1338	GAAGGTGATCGGGACGTTGAT		
NM_174744.2	*MMP9*	F.1169	GCCCGGATCAAGGATACAGC	128	97.3
		R.1296	GGGGTGCTCCTCTGTGAATC		
NM_174182.1	*SELL*	F.588	CTCTGCTACACAGCTTCTTGTAAAC	104	93.5
		R.691	CCGTAGTACCCCAAATCACAGTT		
NM_001037628.2	*SELPLG*	F.123	CTGAGCACGGTGCCATGTTTC	111	94.7
		R.233	CTGGGGCCTTCACAGTTTCA		
NM_174008.1	*CD14*	F.525	TCCGTAACGTATCGTGGACAAC	100	96.8
		R.624	GAGTGTGCTTGGGCAATGTTC		
XM_003586675.4	*LCN2*	F.749	CCAGTGAGCCTGCACCTTTG	141	98.5
		R.889	TATTTAGCAGGCAAGGCAGGG		
NM_001113298.2	*MPO*	F.1311	AGCCATGGTCCAGATCATCAC	105	97.6
		R.1415	ACCGAGTCGTTGTAGGAGCAGTA		
NM_174615.2	*SOD1*	F.256	GGCTGTACCAGTGCAGGTCC	101	99.8
		R.356	GCTGTCACATTGCCCAGGT		
NM_201527.2	*SOD2*	F.620	TGTGGGAGCATGCTTATTACCTT	95	96.7
		R.714	TGCAGTTACATTCTCCCAGTTGA		
NM_174197.2	*TLR2*	F.3182	CCATGTCTGGAGAGGGTGTT	102	94.9
		R.3283	GGGGACACAAAACAGCACTT		
NM_174178.2	*SDHA*	F.1563	CTGAAGCAGGTTTCAACACG	130	95.2
		R.1544	GTTGTCCTCCTCCATGTTCC		
NM_173925.2	*IL8*	F.350	GTGAAGAGAGCTGAGAAGCAAG	150	94.6
		R.499	CACCAGACCCACACAGAACAT		
NM_173979.3	*ACTB*	F.258	ACCAACTGGGACGACATGGA	149	92.6
		R.406	GTCTCGAACATGATCTGGGTCAT		
NM_174198.6	*TLR4*	F.102	GCTGTTTGACCAGTCTGATTGC	102	95.7
		R.203	GGGCTGAAGTAACAACAAGAGGAA		
NM_001078159.1	*LYZ*	F.281	AAAGCAGTTAACGCCTGTCGTAT	122	93.8
		R.402	CATGCCACCCATGCTTTAATG		
NM_001102219.1	*NLRP3*	F.69	CTTTCTGGACTCTGACCGGG	149	92.4
		R.217	ATTGAGGTGCAGCCCTTCTG		
NM_001040555.1	*IRAK1*	F.950	CCTCAGCGACTGGACATCCT	103	93.8
		R.1052	GGACGTTGGAACTCTTGACATCT		
NM_174093.1	*IL1B*	F.120	ATTCTCTCCAGCCAACCTTCATT	100	98.6
		R.256	TTCTCGTCACTGTAGTAAGCCATCA		
NM_001077402.1	*CD16*	F.252	CAGGACAGTGGCGAGTACAAGT	100	95.6
		R.351	GAGCGACCTGGAGCAATAGC		
NM_001014382.2	*MYD88*	F.367	GGAGGACTGCCAAAAGTATATTCT	105	94.4
		R.471	GCCATGTCATTTATCCGAGTTATG		
NM_001113725.2	*S100A8*	F.19	ATTTTGGGGAGACCTGGTGG	124	94.8
		R.142	ACGGCGTGGTAATTCCCTTT		
NM_001101866.2	*IDO1*	F.18	ACTGCAAGAATGGCAGGTGA	125	100.1
		R.142	GGATGAGGTAGGTCCTCCAGT		

Supplemental Table S1. GenBank accession number, primer hybridization position, primer sequence, amplicon size, and amplification efficiency of primer pairs used for qPCR analysis of gene expression in Bos taurus.

**Accession no.**	**Gene**	**Primers[1]**	**Primers (5'-3')**	**(bp)[2]**	**Efficiency (%)**
NM_001192792	*ALOX5*	F.771	GCAGGAAGACCGCATGTTTG	163	97.6
		R.933	GTTCCCTTGCTCGATCTCCT		
NM_174501.2	*ALOX15*	F.1495	AGGCCTGGTGTCGAGATATCA	105	99.4
		R.1599	TGGTCACAAAGTGGCAAAGC		
NM_174091.2	*IL18*	F.366	GACTGTTCAGATAATGCACCCC	126	97.3
		R.491	GTTCTCACAGGAGAGAGTAGAC		
NM_174013.3	*CD44*	F.752	GAAGCACTTCAGGAGGCTACA	125	94.8
		R.876	GCCGTAGTCTCTGGTATCCG		
NM_174674.2	*TNFRSF1A*	F.936	CTGGTGATTGTCTTCGGGCT	104	95.9
		R.1039	TGCCCGCAAATGATGGAGTA		
NM_173923.2	*IL6*	F.190	CCAGAGAAAACCGAAGCTCTCAT	100	97.8
		R.289	CCTTGCTGCTTTCACACTCATC		
NM_174088.1	*IL10*	F.171	GAAGGACCAACTGCACAGCTT	98	100.2
		R.268	AAAACTGGATCATTTCCGACAAG		
NM_001110785.3	*IL6R*	F.570	GCTCTTTCTACGTATTGTCCCTGTGT	100	95.1
		R.669	GGGTCGGGCTGTAGGAGTTT		
XM_592026.7	*CASP1*	F.193	AGTGCTGAACCAGGAGGAGA	188	95.7
		R.380	CAGACTGTGAACCTGAAGTGAG		
NM_174484.1	*VCAM1*	F.1466	ACCAAGAGTTTGGAGAAGACC	152	97.4
		R.1617	CCCTGGGAGCAACATTAACA		
NM_173966.3	*TNFα*	F.174	CCAGAGGGAAGAGCAGTCCC	114	92.6
		R.287	TCGGCTACAACGTGGGCTAC		
NM_174814.2	*YWHAZ*	F.	TGAAAATGAAAGGAGACTACTACCG	84	101.1
		R.	GCTGTGACTGGTCCACAATC		
NM_001102558.2	*CX3CR1*	F.75	CCCAGCCCAGGTGCTCA	175	96.1
		R.249	CAGCAAATTTCCCACCAGGC		
XM_002689143	*PRTN3*	F.222	TGCCTGAACAACTTGAACCC	108	93.2
		R.329	CTCAAACAGGCGACTGATGC		

[1] Primer direction (F – forward; R – reverse) and hybridization position on the sequence. are underlined.

[2] Amplicon size in base pair (bp).
